# Operative Treatment of Fixed Flexion Deformity of Elbow by Anterior Release: A Case Report

**DOI:** 10.31729/jnma.5189

**Published:** 2020-11-30

**Authors:** Abhishek Kumar Thakur, Nabees Man Singh Pradhan, Pramod Devkota, Bidur Gyawali, Prabhav Majgaiyan Pokhrel

**Affiliations:** 1 Department of Orthopedics and Trauma Surgery, Patan Academy of Health Sciences, Lagankhel, Lalitpur

**Keywords:** *anterior release*, *elbow*, *fixed flexion deformity*

## Abstract

A 20-year-old male presented to our outpatient department with stiffness in his right elbow. He gave a history of sustaining a fracture around the same elbow when he was 4 years old. He was treated operatively for the same. In the postoperative period, he did not undergo any physiotherapy. On examination, he had a fixed flexion deformity in his right elbow with a range of motion between 90 and 110 degrees. The X-ray did not show any bony abnormalities and magnetic resonance imaging revealed susceptibility artifacts in the posterior aspect. The elbow was approached anteriorly releasing all the soft tissue contractures. The elbow was immobilized in extension in a plaster cast for 4 weeks. The patient was under regular physiotherapy after plaster removal in the post-operative period. At one year follow up, he has an elbow range of motion between 20 and 120 degrees.

## INTRODUCTION

Elbow stiffness with loss of function is a common disabling problem that usually arises as a complication of trauma, burns, head injury, or in association with degenerative, inflammatory, or hemophiliac arthropathy and congenital malformations. Loss of elbow extension commonly produces a significant functional deficit.^1^ Several conservative measures including static and dynamic splinting, physical therapy, and hinged external distraction have been used to provide a lasting improvement in elbow motion. The most refractory contractures require anterior elbow release.^2^ We present a case where a fixed flexion deformity of the elbow was treated operatively by anterior release procedure.

## CASE REPORT

A 20-year-old male presented to our outpatient department (OPD) with stiffness in his right elbow. He gave a history of sustaining a fracture around the same elbow when he was 4 years old. He was treated operatively for the same. In the postoperative period, he did not undergo any physiotherapy.

**Figure 1:**
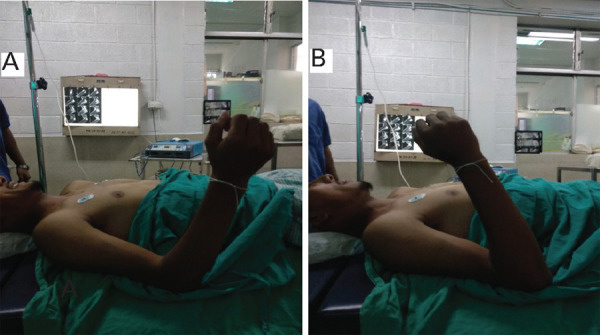
Figure 1 A, B. Pre-operative RoM of the elbow. (A) Full extension, (B) Full flexion.

On examination, he had a fixed flexion deformity in his right elbow with a Range of Motion (RoM) between 90 and 110 degrees (20 degrees free RoM) (Figure 1). The X-ray did not show any bony abnormalities and MRI revealed susceptibility artifacts in the posterior aspect. As this was a case of chronic stiffness, other non-operative treatment options were not considered and he was planned for surgery.

**Figure 2:**
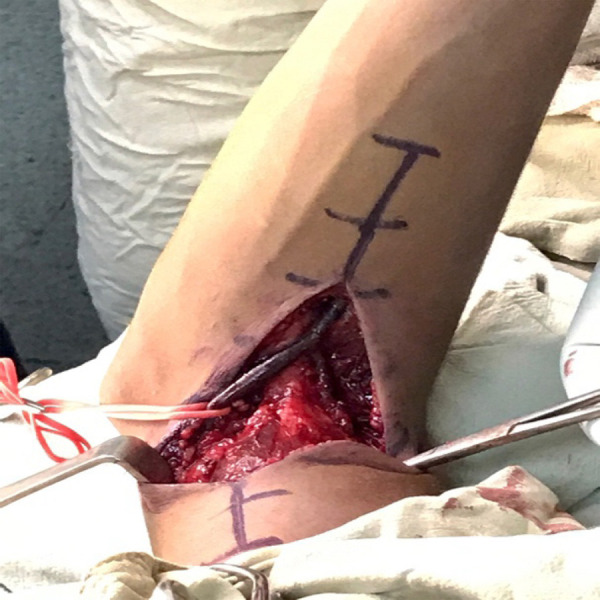
Intra-operative image showing the anterior approach to the elbow.

The elbow was approached anteriorly releasing all the soft tissue contractures (Figure 2). Anterior capsulotomy as well as the release of the anterior portion of the collateral ligaments were performed. The elbow had full extension prior to leaving the operating room.

Surgery was done under tourniquet control, and the wound was closed primarily.

The elbow was immobilised in extension in a plaster cast for 4 weeks. The patient was under regular physiotherapy after plaster removal in the post-operative period. The physiotherapy included gradual, active as well as passive range of motion exercises.

**Figure 3:**
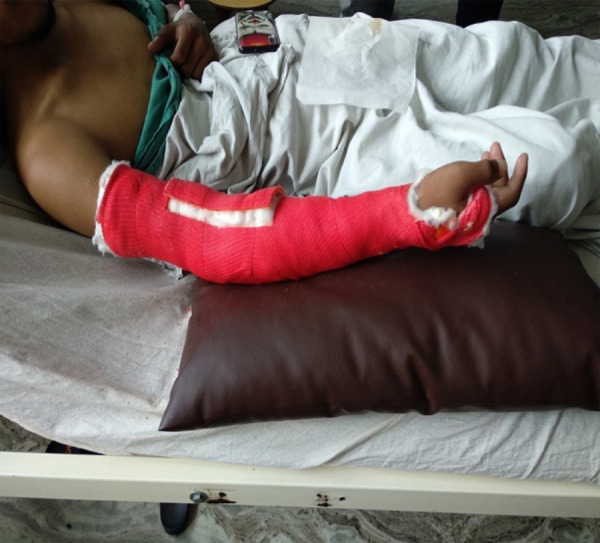
Post-operative image with an elbow in a plaster cast.

**Figure 4 A, B.**
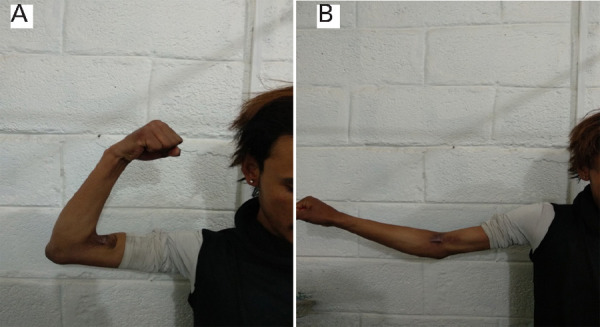
One year post-operative RoM of elbow (A) Full flexion, (B) Full extension.

At one year follow up, he has an active elbow range of motion between 20 and 120 degrees (100 degrees free RoM) (Figure 4).

## DISCUSSION

Elbow contractures can be classified as extrinsic or intrinsic according to the underlying etiology. Extrinsic contractures involve the peri-articular soft-tissues with a normal or near-normal articular surface. Intrinsic factors include disruption of the normal articular surface, osteophytes, intra-articular loose bodies, and secondary osteoarthritis.^3^

When non-operative treatments such as static or dynamic splinting fail, then surgery is often considered. Many surgical techniques have been described for established contractures with significant functional impairment. These include manipulation-under-anesthesia; arthroscopic release; open capsulectomy via anterior, posterior, medial, lateral, or combined approaches.^1^

There have been several series dealing with the results of the anterior release of post-traumatic elbow flexion contractures which have addressed operative techniques such as complete anterior capsulectomy with lengthening of the biceps tendon and/or brachialis myotomy to the more limited anterior release of the capsule (Glynn, 1976; Urbaniak, 1985). The results have been encouraging, but residual contracture and failure to maintain correction have been frequent problems.^4,5^ In our case, we performed anterior capsulotomy and release of the anterior portions of the collateral ligaments. Biceps tendon lengthening or brachialis myotomy was not done.

Breen, et al. has described the use of continuous passive motion therapy in the post-operative period for decreasing the residual contracture.^2^ Our case has achieved an 80-degree free RoM improvement at one year follow up by gradual physiotherapy including active and passive range of motion exercises.

Hence, long-standing fixed flexion deformities, flexion contractures, and stiffness of the elbow can be treated operatively by anterior release, safely, and with optimum results. A regular post-operative physiotherapy program is a must for the best results.
